# Triggering HIV polyprotein processing by light using rapid photodegradation of a tight-binding protease inhibitor

**DOI:** 10.1038/ncomms7461

**Published:** 2015-03-09

**Authors:** Jiří Schimer, Marcela Pávová, Maria Anders, Petr Pachl, Pavel Šácha, Petr Cígler, Jan Weber, Pavel Majer, Pavlína Řezáčová, Hans-Georg Kräusslich, Barbara Müller, Jan Konvalinka

**Affiliations:** 1 Institute of Organic Chemistry and Biochemistry, Academy of Sciences of the Czech Republic, Gilead Sciences and IOCB Research Center, Flemingovo n.2, 166 10, Prague 6, Czech Republic; 2 Department of Biochemistry, Faculty of Science, Charles University in Prague, Hlavova 8, 128 43, Prague 2, Czech Republic; 3 Department of Infectious Diseases, Virology, University Hospital Heidelberg, Im Neuenheimer Feld 324, 69120 Heidelberg, Germany; 4 Molecular Medicine Partnership Unit, Heidelberg, Germany

## Abstract

HIV protease (PR) is required for proteolytic maturation in the late phase of HIV replication and represents a prime therapeutic target. The regulation and kinetics of viral polyprotein processing and maturation are currently not understood in detail. Here we design, synthesize, validate and apply a potent, photodegradable HIV PR inhibitor to achieve synchronized induction of proteolysis. The compound exhibits subnanomolar inhibition *in vitro*. Its photolabile moiety is released on light irradiation, reducing the inhibitory potential by 4 orders of magnitude. We determine the structure of the PR-inhibitor complex, analyze its photolytic products, and show that the enzymatic activity of inhibited PR can be fully restored on inhibitor photolysis. We also demonstrate that proteolysis of immature HIV particles produced in the presence of the inhibitor can be rapidly triggered by light enabling thus to analyze the timing, regulation and spatial requirements of viral processing in real time.

## Abstract (web summary)

The study of HIV proteolysis during maturation and replication can be difficult since different steps in these processes occur simultaneously. Here, the authors present a photolabile HIV protease inhibitor which can be deactivated by light irradiation, allowing synchronized induction of viral maturation.

## Graphical abstract

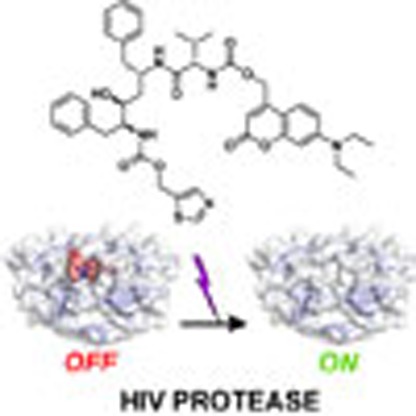

HIV-1 protease (HIV-1 PR) is among the best-studied enzymes in biochemistry. This 99 amino acid long homodimeric aspartic PR plays a pivotal role in the viral replication cycle^1^. PR is synthesized as part of the viral Gag-Pol polyprotein. Approximately 125 molecules of Gag-Pol co-assemble at the plasma membrane with ~2,500 molecules of the main structural polyprotein Gag to create an immature virion. In the assembled immature particle, the PR domain of Gag-Pol cleaves Gag and Gag-Pol at nine distinct sites to create the mature, functional subunits. Proteolytic processing results in a dramatic rearrangement of the particle core termed maturation, which is a prerequisite for HIV-1 infectivity. Consequently, inhibitors of HIV-1 PR are powerful virostatics. Due to major efforts from both academia and industry 10 specific HIV-1 PR inhibitors are currently available for antiretroviral therapy (for review, see ref. 2).

While the structure and the enzymatic properties of HIV-1 PR *in vitro* are well characterized, key questions concerning proteolytic maturation remain unanswered. Virological studies from many groups indicate that the maturation process needs to be tightly controlled. Not only inhibition, but also premature activation of PR is detrimental for virus replication^3^, and blocking or even partially inhibiting processing at one of the cleavage sites strongly reduces HIV-1 infectivity^4^. According to current understanding, Gag proteolysis occurs when the polyprotein has already assembled into a tight hexameric lattice, but it is unclear what prevents premature proteolysis and how PR is activated once the immature virion has been assembled^5^. Furthermore, the sequence, timing, and topology of cleavage events during particle maturation remain largely unclear. The key obstacle in dissecting this complex process is the asynchronous formation of mature HIV-1 particles in tissue culture, since any virus population harvested from culture media constitutes an ensemble of particles in different stages of polyprotein processing and maturation. Overcoming this fundamental obstacle requires an experimental tool for triggering HIV-1 PR activity at a defined moment, thus inducing and synchronizing the viral maturation process.

Several approaches can in principle be used to achieve synchronization. Temperature-sensitive PR mutants have been developed to analyze individual steps of the replication cycle of picorna and other viruses^6^. However, attempts to prepare temperature-dependent mutants of HIV-1 PR have met with limited success. Although several HIV-1 PR mutants with temperature-dependent differences in proteolytic activity have been reported, none of these allowed switching from a non-active to an active enzymatic state, which would be required to trigger HIV-1 maturation^7^,^8^.

Alternatively, one may induce proteolysis by wash-out of a specific PR inhibitor from immature particles produced in the presence of the inhibitor. We have recently explored this strategy by systematic testing of a panel of available and experimental PR inhibitors and found that PR activation can indeed be accomplished by inhibitor wash-out, provided that inhibitors with a high off-rate are used^9^. With a half-time of 4-5 h, the kinetics of proteolysis were slow, however, and morphologically mature virus particles and virus infectivity were not recovered^9^. Accordingly, inhibitor wash-out does not appear to trigger functional maturation and more efficient and faster induction of proteolysis inside the immature virion may be needed.

A possible way to overcome this limitation is the use of caged compounds that are released on irradiation with light of a specific wavelength. The release of effector molecules by light-induced cleavage of inactive precursors is well-established in chemical biology. Following pioneering studies describing photocaged cAMP and ATP^10^,^11^, photocaged small molecules acting as secondary messengers, for example, calcium^12^ and nitric oxide^13^, as well as caged hormones^14^,^15^, neurotransmitters^16^,^17^, nucleic acids^18^,^19^ and diacylglycerols^20^ were developed. Whole proteins have also been caged to analyze signalling and other regulatory events in the cell (for example, refs 21, 22; for recent reviews covering caged small molecules, see refs 23, 24, 25).

To trigger the activity of an enzyme in the absence of a specific small molecule activator would require a caged version of the enzyme of interest. However, caging of a large biomolecule presents a major technical challenge. Furthermore, the caged protein must be delivered into the cell (for example, by microinjection), and would compete with the endogenously expressed protein^26^. In the specific case of HIV-1 PR, the enzyme is part of a polyprotein which needs to be incorporated into the nascent virus particle, rendering this strategy not feasible.

An alternative to protein caging is the use of a photolabile enzyme inhibitor that could be inactivated by light, to trigger enzyme activity. An effective photodegradable enzyme inhibitor is characterized by a substantial decrease in inhibitory activity on photolysis, and a few examples for this strategy have been published. Li *et al*.^26^ connected two peptidic inhibitors of two distinct domains of Src kinase via a photodegradable linker. Separation of the bivalent inhibitor into two compounds, each binding the target with lower affinity, reduced inhibitory potency by ~50-fold. Porter *et al*.^27^ developed a mechanism-based approach to analyze activation of a serine PR. Photolysis of a covalent adduct in the active site of the PR led to PR activation.

The wealth of structure-activity data accumulated on purified HIV-1 PR and its inhibitors renders HIV-1 polyprotein processing and maturation an excellent target for the development of a specific photoinactivatable inhibitor. We thus set out to develop a method to activate HIV-1 maturation by photodegradation of a specific and potent PR inhibitor. Here we describe the design, synthesis, validation and application of a subnanomolar HIV-1 PR inhibitor that is cleaved on irradiation by 405-nm light. After irradiation, the inhibitor’s potency decreases by 4 orders of magnitude, leading to nearly full restoration of enzyme activity and to rapid induction of polyprotein processing in assembled HIV-1 particles in tissue culture.

## Results

### Kinetic analysis of inhibitor and its photodegradation product

**Figure 1:**
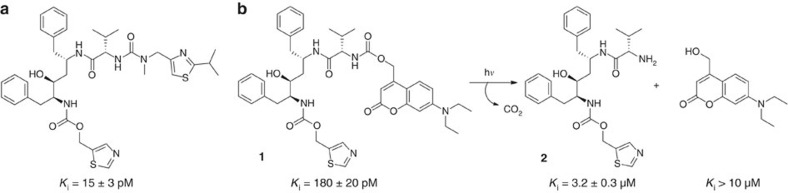
A photolabile inhibitor of HIV-1 PR and its degradation triggered by light. (**a**) HIV-1 protease inhibitor Ritonavir; (**b**) Photodegradable inhibitor of HIV-1 PR (compound **1**) and products resulting from photolysis (compound **2** and coumarin derivative). Inhibition constants determined as in Fig. 3a are shown for each compound.

The design of a photoinactivable inhibitor of HIV-1 PR (**1**) was based on the structure of the PR inhibitor ritonavir (RTV; Fig. 1a)^28^. Compound **1** contains a photolabile 7-diethylamino-4-(hydroxymethyl)coumarin group connected via a carbamate linker to a RTV fragment (Fig. 1b), and is a tight-binding inhibitor of HIV-1 PR, displaying subnanomolar inhibition potency (*K*_i_=170±20 pM, for detailed synthesis see Supplementary Fig. 1). The degradation fragment **2** displayed only weak inhibitory activity, with a *K*_i_ value of 3.2±0.3 μM (Fig. 1b). The other product of photolysis, the coumarin group, did not show any inhibitory activity at a concentration of 10 μM. The identities of the photodegradation fragments of compound **1** were confirmed by independent measurement using analytical high-performance liquid chromatography (HPLC; Supplementary Fig. 2).

### Determination of binding mode into HIV-1 PR

To investigate the binding modes of compounds **1** and **2**, we co-crystallized both with HIV-1 PR and determined the corresponding structures at resolutions of 1.6 Å and 1.4 Å, respectively. Both crystals were of identical space group (*P*6_1_), and contained one PR dimer in the asymmetric unit. The structures were refined with two inhibitor molecules bound in alternative orientations related by 180° rotation with 50% relative occupancy. The quality of the electron density map for residues 35–60 for 35–45 for HIV-1 PR-**1** and HIV-1 PR-**2** complex, respectively, was limited, suggesting that these regions of flaps are partially disordered. The electron density for the remaining part of the protein as well as compounds bound to the active site were of good quality enabling unambiguous modelling (Supplementary Fig. 3).

**Figure 2:**
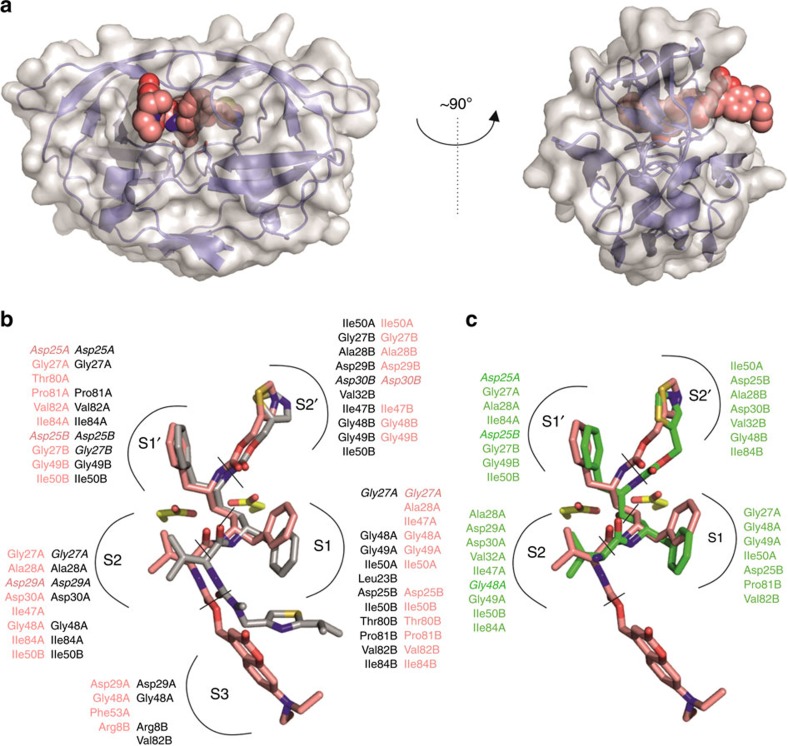
Comparison of binding mode of compounds 1 and 2 to HIV-1 PR. (**a**) Two views of the HIV-1 PR-**1** complex (PDB code 4U7Q). The protein is shown in cartoon representation with a transparent surface, while the inhibitor atoms are represented by spheres. The coumarin moiety protrudes from the enzyme active site cavity. (**b**) Superposition of **1** (pink carbon atoms) and RTV (ritonavir; grey carbon atoms) bound in the HIV-1 PR active site. (**c**) Superposition of **1** with **2** (in green, PDB code 4U7V) bound to HIV-1 PR (PDB code 1HXW (ref. 28)). (**b**,**c**) Residues interacting with **1**, **2** and RTV are indicated in the corresponding colours for individual enzyme subsites. Residues forming polar interactions are highlighted in bold italics. To identify non-polar interactions, the cut-off for distance between any atom of residue and any atom of inhibitor was 4 Å. For polar interaction, the cut-off for distance between hydrogen bond donor and acceptor was 3.5 Å. Active site aspartates are shown in stick representation.

The crystal structures revealed that compounds **1** and **2** both occupy enzyme subsites S2, S1, S1′ and S2′. In addition, compound **1** interacted with the S3 enzyme subsite through the coumarin moiety. The extent of interactions with S3 residues was rather limited, however, and the coumarin moiety protruded from the enzyme active site cavity (Fig. 2a). The suboptimal interaction in the S3 pocket, compared with the interaction of the P3 moiety of RTV, is likely to contribute to the 10-fold difference in *K*_i_ values for RTV and compound **1**. Binding of the P2′, P1′ and P2 substituents of compound **1** was similar to that of RTV (Fig. 2b), whereas differences were observed for interactions in the S1 and S2 pockets. In the HIV-1 PR-compound **1** complex, the carbamate between the coumarin moiety and the inhibitor formed a hydrogen bond with the Asp29A side chain, similar to the interaction of the corresponding group of RTV.

Interestingly, the positions and conformations of moieties common to compound **1** and **2** as well as their interactions within the S2 to S2′ subsites were quite different (Fig. 2c). The largest difference was observed in the P1 and P2′ moieties, where the extent of interactions was much lower for compound **2** compared with compound **1**. This might provide a structural explanation for the observation that compound **2** displayed a low inhibitory activity against HIV-1 PR, considering that it occupies the S2 to S2′ enzyme binding subsites. In addition, compound **2** differs from compound **1** in that it has a free terminal amine group, which is charged at lower pH and likely repulsed from the enzyme cavity. In support of this hypothesis, acetylation of the free amine of compound **2** led to a major increase in inhibitory activity.

### Reactivation of HIV-1 PR by photolysis of compound 1

**Figure 3:**
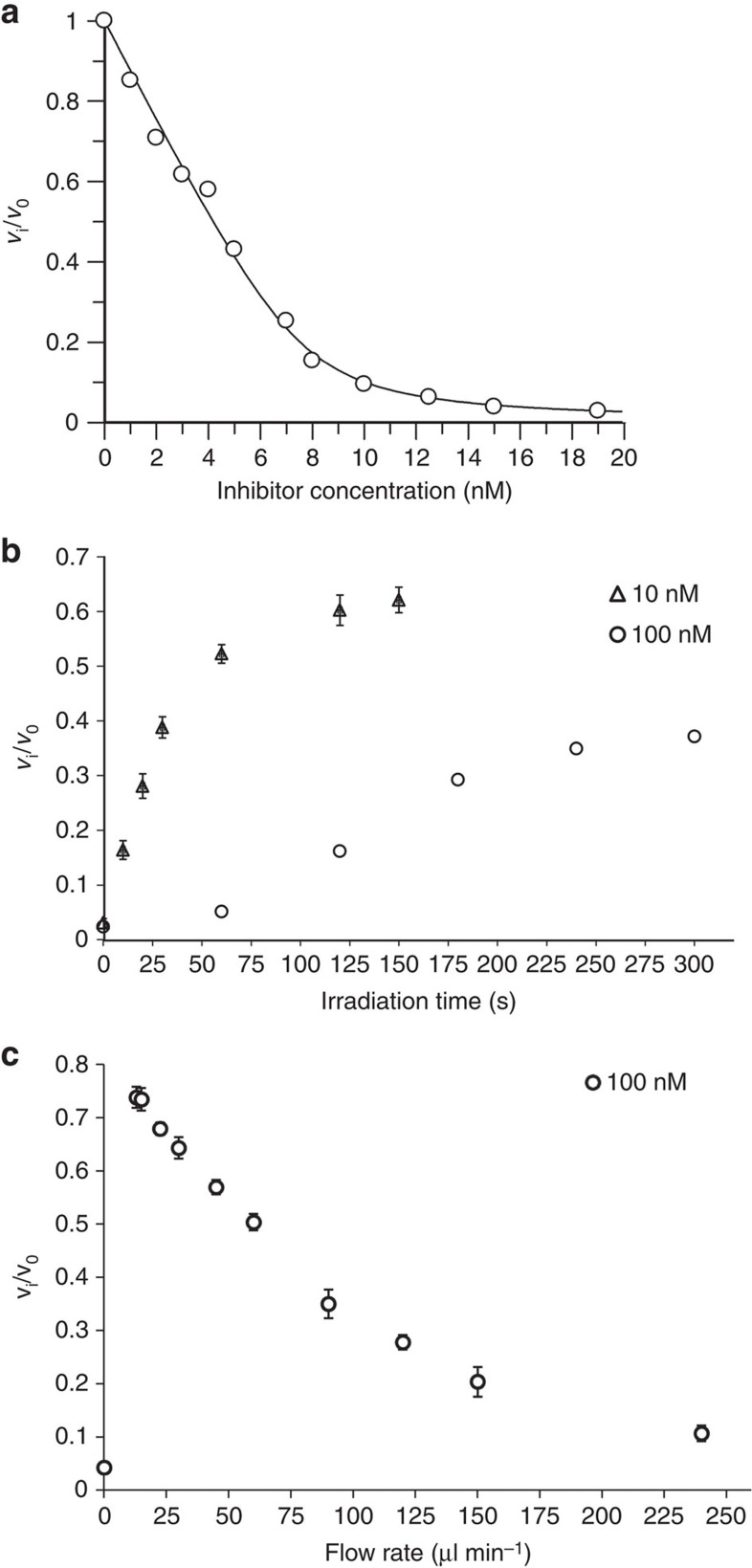
Kinetic analysis of HIV-1 PR reactivation by inhibitor photodegradation. (**a**) A non-linear fit of Morrison equation of inhibition of HIV-1 PR by compound **1**. The activity of purified recombinant HIV-1 PR was determined *in vitro* as described in the experimental section in the presence of the indicated inhibitor concentrations. Two independent experiments yielded very similar results; (**b**,**c**) Reactivation of purified recombinant HIV-1 PR in buffer (100 mM sodium acetate, 300 mM NaCl, 4 mM EDTA, pH 4.7) by photodegradation of **1** using either the cuvette set up (**b**) or the capillary set up (**c**): (**b**) Purified recombinant HIV-1 PR (8 nM) incubated with compound **1** at the indicated concentrations was irradiated with two 405 nm lasers (combined output of 300 mW) for various time intervals. The PR activity was then measured using a chromogenic substrate. The plot shows relative PR activity as a function of time. (**c**) Purified recombinant HIV-1 PR (160 nM) incubated with 2 μM compound **1** was pumped at different flow rates through a thin glass capillary onto which two 405 nm lasers (combined output of 300 mW) were focused (for set up see Supplementary Fig. 4). Relative PR activity was determined as in **b** after 20-fold dilution into cleavage buffer using the same chromogenic substrate (for details, see Experimental section) and plotted against the flow rate of the sample through the capillary. Flow rate 0 represents a non-irradiated sample. The graph shows mean values and s.d. from three independent experiments.

To evaluate the efficacy of photodegradation, we analyzed the restoration of HIV-1 PR activity that had been inhibited by compound **1** (Fig. 3a) on irradiation at 405 nm. For irradiation, two lasers with outputs of 130 mW and 170 mW, respectively, were used in parallel. First, we tested a standard cuvette set up, in which 8 nM purified recombinant HIV-1 PR (final concentration of compound **1**: 10 or 100 nM; Fig. 3b) in 1 ml cleavage buffer was irradiated. At the lower concentration, irradiation led to a significant restoration of enzyme activity (65% compared with the uninhibited enzyme reaction). At the higher concentration of compound **1**, however, PR reactivation was limited even after prolonged irradiation (only 35% activity after 5 min of irradiation). We reason that the product of photodegradation (compound **2**) interferes with degradation of compound **1** in this set up, because the absorption spectrum of compound **2** is almost identical to that of compound **1**. Therefore, we investigated a number of alternative irradiation set ups, and obtained optimal results when the inhibitor solution was irradiated while being pumped through a thin glass capillary at which the two lasers were focused (Fig. 3c). This set up ensured homogeneous irradiation throughout the sample and prevented absorption of light by the released coumarin product. At a flow rate of 15 μl min^−1^, up to 75% of original PR activity was recovered, despite an inhibitor concentration 4 orders of magnitude above its *K*_i_ value (*K*_i_=170 pM, inhibitor concentration 100 nM). Considering that compound **1** is a tight-binding inhibitor (see Fig. 3a) and 75% of the PR activity was restored, we estimate that up to 98% of compound **1** was degraded.

### Reactivation of HIV-1 PR inside immature virions

**Figure 4:**
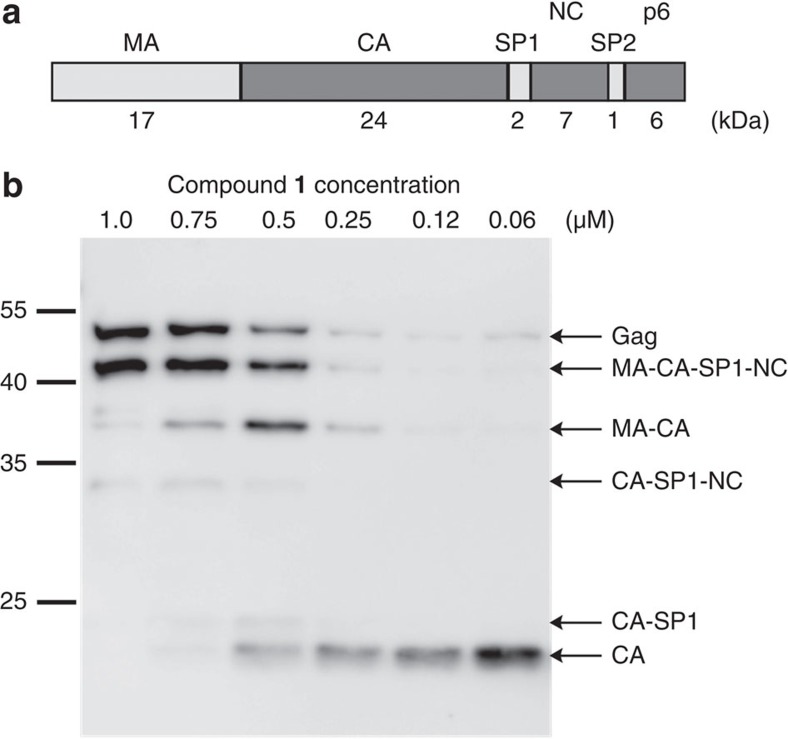
Inhibition of HIV-1 Gag processing by compound 1. (**a**) Schematic representation of the 55 kDa HIV-1 Gag polyprotein and its cleavage products. (**b**) Inhibition of HIV-1 Gag processing by compound **1.** HIV-1 particles were produced in HEK293T cells in the presence of the indicated inhibitor concentrations. The experiment was performed in duplicate and a representative result is shown. Molecular mass standards are shown on the left; Gag and its respective cleavage products are identified on the right. CA, capsid; MA, matrix; NC, nucleocapsid; p6, p6 protein; SP1, spacer peptide 1; SP2, spacer peptide 2.

Based on these results, we analyzed whether photodegradation of compound **1** can be used to trigger HIV-1 PR activity inside intact immature HIV-1 particles produced in tissue culture in the presence of the inhibitor. PR-mediated cleavage of the Gag polyprotein yields the mature structural proteins and two small spacer peptides (Fig. 4a), and different rates of cleavage at individual sites result in the generation of characteristic processing intermediates^29^. First, we assessed the inhibitory potency of compound **1** on HIV-1 Gag polyprotein processing in virus producing cells. For this, particles were purified from the supernatant of HEK293T cells transfected with an HIV-1 proviral plasmid and incubated in the presence of various concentrations of compound **1**. Gag processing was efficiently inhibited with ~50% reduction in Gag cleavage at ~500 nM compound **1** (Fig. 4b). Previous studies had indicated that infectivity is severely impaired at concentrations where polyprotein processing is only inhibited to a minor extent^30^,^31^. Accordingly, compound **1** inhibited HIV-1 replication in the MT-4T-cell line with an EC_50_ of 8.1 nM, although almost no effect on Gag processing could be seen at this concentration (Supplementary Fig. 5). Compound **1** was soluble and noncytotoxic at a concentration of 2 μM in 0.5% DMSO (CC_50_=7.3 μM for MT4, CC_50_>50 μM for HEK293T cells; for more detailed information see Supplementary Chapter 5).

**Figure 5:**
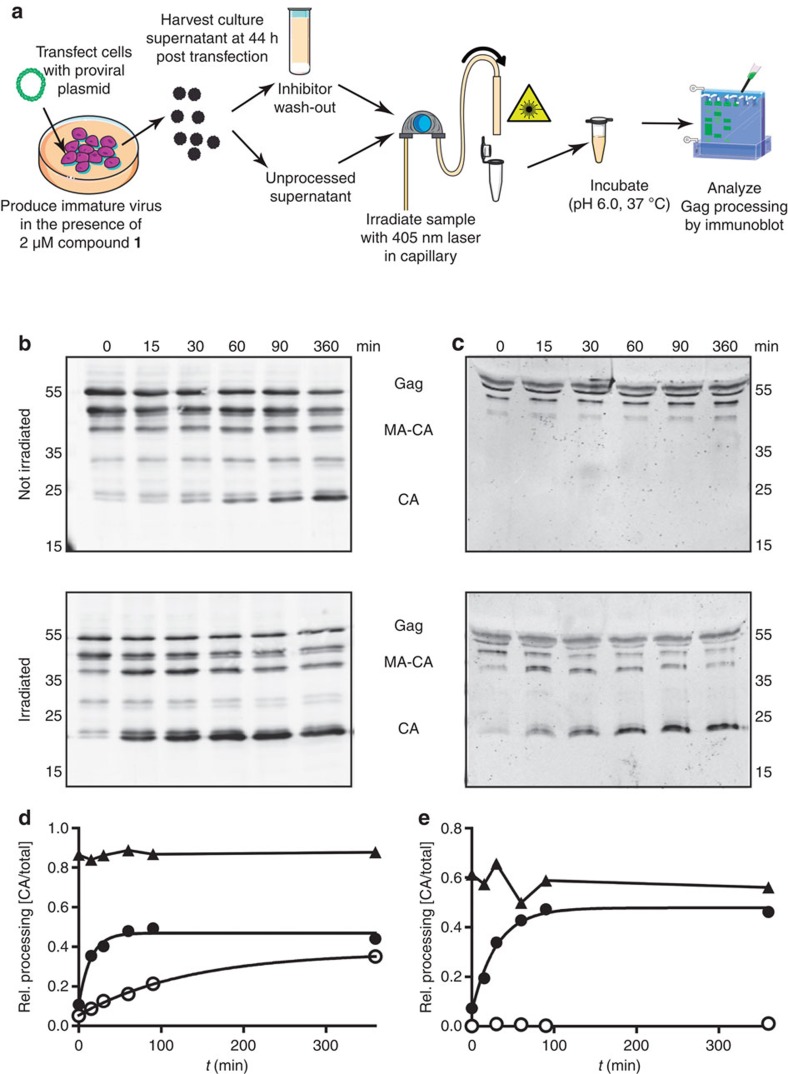
Photoinduced Gag processing in the context of the assembled virion. (**a**) Schematic illustration of the irradiation experiment to trigger HIV-1 maturation using photoinactivation of compound **1**. HEK293T cells were transfected with a proviral HIV-1 plasmid and particles were produced in the presence of 2 μM compound **1**. At 44 h post transfection, tissue culture supernatant was harvested and either subjected to ultracentrifugation (**b**,**d**) or used directly (**c**,**e**). In both cases, samples were then pumped through the capillary set up shown in Supplementary Fig. 4 either with or without ultraviolet irradiation. Subsequently, samples were incubated for various lengths of time. (**b**,**c**) Immunoblot analysis of Gag processing products. Samples were separated by SDS–PAGE, and products of Gag processing were detected by quantitative immunoblot (LiCor) using antiserum raised against recombinant HIV-1 CA. The figures show samples incubated for the indicated times without prior irradiation or following irradiation, respectively. Positions of Gag-derived proteins are indicated. (**d**,**e**) Quantitative analysis of the experiments shown in **b** or **c**, respectively. Anti-CA reactive bands from the immunoblots shown and from corresponding blots from irradiated mature control virus produced in the absence of inhibitor (not shown here) were quantified using Image Studio Light. The graphs show the proportion of mature CA relative to the sum of all anti-CA reactive bands in the respective lane. Filled triangles, irradiated control virus; open circles, inhibitor-treated virus, not irradiated; filled circles, inhibitor-treated virus, irradiated. Curves through data from inhibitor-treated samples represent fits to a single exponential equation. The results are representative of several independent experiments with a slight variation in the half-time of Gag polyprotein processing between 20 and 30 min. CA, capsid; MA, matrix.

Having established conditions for the inhibition of HIV-1 polyprotein processing by compound **1**, we proceeded to prepare immature particles for *in situ* activation of PR. For this, virus particles were produced in transfected HEK293T cells grown in the presence of 2 μM inhibitor. The experimental set up is schematically illustrated in Fig. 5a. Particles collected from inhibitor-treated cells were either subjected to inhibitor wash-out by two subsequent ultracentrifugation steps (Fig. 5b), or only filtered to remove cell debris (Fig. 5c). In both cases, samples were then either irradiated (405 nm) in a glass capillary (Fig. 5b,c, bottom panels) or passed through the capillary in the absence of irradiation (Fig. 5b,c, top panels).

Wash-out of compound **1** in the absence of irradiation resulted in slow PR activation and Gag processing (Fig. 5b, top), with kinetics closely resembling those observed in our previous study (Fig. 5d)^9^. In contrast, mature CA was produced much more rapidly in the irradiated sample (Fig. 5b, bottom), yielding ~30% mature CA within 15 min (Fig. 5d). Gag processing remained incomplete, however, with ~50% unprocessed or partially processed products remaining at 6 h of incubation. Direct irradiation of particle-containing culture medium without prior ultracentrifugation allowed us to process samples much more rapidly, thereby protecting sample integrity and PR activity. In this set up, no induction of proteolysis was observed in the absence of irradiation even after prolonged incubation (Fig. 5c, top). In contrast, irradiation of culture medium without any inhibitor removal resulted in rapid PR activation and virtually complete Gag processing (Fig. 5c, bottom). The irradiated sample exhibited a level of Gag processing comparable to that observed for an uninhibited control virus after ~2 h of incubation, yielding an apparent half-time of ~20 min for Gag processing (Fig. 5e).

## Discussion

This report demonstrates that a photodestructible inhibitor can be used to trigger HIV-1 PR activation and polyprotein processing *in situ* inside the assembled immature virus. This was accomplished by design, synthesis and validation of a photolabile tight-binding inhibitor of HIV-1 PR (*K*_i_=170±20 pM) that demonstrates a 4-order-of-magnitude loss of potency on irradiation with a 405 nm laser. The inhibitor thus served as a photocage for HIV-1 PR activity and allowed initiating HIV-1 polyprotein processing within the assembled virion in a controlled manner. Analysis of Gag processing kinetics under these conditions revealed that the mature CA subunit was released with a half-time of ~20–30 min, which is substantially faster than the half-time of 4–5 h observed using an optimized inhibitor wash-out strategy^9^. The direct comparison between wash-out and photodestruction of the newly described inhibitor (Fig. 5) clearly showed that much more rapid activation is accomplished by photodestruction. Furthermore, processing rates were not enhanced by including a wash-out step prior to irradiation, demonstrating the effectiveness of photodestruction.

At first glance, the half-time of 20–30 min measured in the experiments shown in Fig. 5 appears rather slow. It needs to be considered, however, that the experiments with immature virus do not directly measure the kinetics of PR activation by photodestruction, as the biochemical experiments shown in Fig. 3, but rather reflect the production of mature CA by processing of the Gag polyprotein assembled in a tight hexagonal lattice. Although HIV-1 proteolytic maturation presumably involves only the viral PR and its substrates Gag and Gag-Pol in the relatively defined environment of the virus particle, the reaction entails at least 66 distinct substrates, intermediates or products, and numerous competing intermolecular interactions occurring simultaneously^32^. Arrangement of the substrate in a multimeric lattice presents further constraints that likely reduce processing rates. The time course of this complex reaction in the virus is currently unknown, and our current study provides an upper time limit for HIV-1 polyprotein processing. Estimates for the period required for polyprotein processing in retroviruses in the literature are based almost exclusively on indirect and very limited evidence and range from a few minutes up to several hours^33^ for completion of proteolytic maturation. Modelling based on simplified assumptions from *in vitro* data yielded an estimate of 30 min for completion of HIV proteolysis^32^, which would be in good agreement with our results. We want to emphasize, however, that our results provide an upper limit for the half-time of Gag proteolysis, assuming instantaneous and complete photodestruction and concomitant PR activation inside the immature particle. Conceivably, authentic polyprotein processing may be even faster, while the slower rates of Gag proteolysis reported in a previous study^33^ are clearly inconsistent with our results.

The possibility to trigger HIV-1 PR activity by light in precisely defined time and space enabled us to induce HIV-1 polyprotein processing inside the native immature virion, and to analyze the timing, regulation, spatial requirements and kinetics of Gag proteolysis in real time. This system now provides the opportunity for a targeted analysis of HIV-1 maturation, which should eventually lead to an understanding of the dynamics of this crucial step in the HIV-1 life cycle. We suggest that a similar approach, that is, design of specific photolabile inhibitors that can be photolysed to inactive products, thus triggering enzymatic activity, can be used for photocaging of other regulatory PRs to analyze their roles *in situ.*

## Methods

### Chemical synthesis

The synthesis of all intermediates and their full chemical analyses are described in the Supplementary Information. All compounds tested in biochemical assays were of at least 99% purity. All peaks in NMR spectra for all compounds were assigned using standard 2D NMR techniques (COSY, HMBC, HSQC).

Compound **1**: Compound **2** (20 mg, 31.3 μmol, 1.0 equiv.) was dissolved in 0.5 ml tetrahydrofurane along with 16 μl +N,N-Diisopropylethylamine (93.9 μmol, 3.0 equiv.). (7-(Diethylamino)-2-oxo-2H-chromen-4-yl)methyl(2,5-dioxopyrrolidin-1-yl) carbonate (13.5 mg, 34.4 μmol, 1.1 equiv. (for preparation, see Supplementary Information) was added in one portion, and the reaction was stirred overnight. The crude product obtained after removal of all volatiles was purified on preparative scale HPLC (gradient 50–100% acetonitrile in 30 min. *R*_*t*_=16 min). Yellow powder was obtained on lyophilization (10 mg, isolated yield=35%). Analytical HPLC (gradient 2–100% in 30 min, flow rate 1 ml min^−1^; *R*_*t*_=25.5 min). ^1^H NMR (500 MHz, DMSO*-d6*):*δ* 9.05 (d, *J*=0.8, 1H, N*-CH*-S), 7.86 (q, *J*=0.8, 1H, S-C-*CH*-N), 7.79 (d, *J*=8.7, 1H, CH-*NH*-Val), 7.45 (d, *J*=9.7, 1H, Val-*NH*-COO), 7.45 (d, *J*=9.1, 1H, C-*CH*-CH-C-N-Et_2_), 7.23-7.07 (m, 10H, 2 × *Ph*), 6.91 (d, *J*=9.4, 1H, *NH*-CH-CH-OH), 6.68 (dd, *J*=9.1, 2.5, 1H, C-CH-*CH*-C-N-Et_2_), 6.55 (d, *J*=2.5, 1H, C-*CH*-C-N-Et_2_), 6.08 (t, *J*=1.3, 1H, O-C(O)-*CH*-C), 5.25 and 5.25 (2 × dd, *J*=16.1, 1.3, 2 × 1H, O-*CH*_*2*_-coumarin), 5.11 and 5.16 (2 × d, *J*=13.0, 2 × 1H, O-*CH*_*2*_-thiazole), 4.15 (bm, 1H, *CH*-NH-Val), 3.80 (bm, 1H, NH-*CH*-CH-OH), 3.78 (dd, *J*=9.2, 7.3, 1H, NH-C(O)-*CH*(iPr)-NH), 3.59 (td, *J*=6.7, 2.1, 1H, NH-CH-*CH***-**OH), 3.42 (q, *J*=7.0, 4H, *CH*_*2*_-CH_3_), 2.69 (bdd, *J*=13.5, 4.9, 1H, *CH*_*2*_**-**CH-NH-Val), 2.67 (bd, *J*=7.6, 2H, *CH*_*2*_-CH-NH-C(O)o-thiazole), 2.59 (bdd, *J*=13.5, 8.0, 1H, *CH*_*2*_**-**CH-NH-Val), 1.85 (dsept, *J*=7.3, 6.8, 1H, -*CH*(CH_3_)_2_), 1.45 (m, 2H, OH-CH-*CH*_*2*_-CH-NH), 1.11 (t, *J*=7.0, 6H, CH_2_-*CH*_*3*_), 0.76 and 0.79 (2 × d, *J*=6.8, 6H, -CH(*CH*_*3*_)_2_). ^13^C NMR (125.7 MHz, DMSO*-d6*):*δ* 161.01 (NH*-(O)CVal***-**NH), 155.92 (O-*C*-CH-C-N-Et_2_), 155.79 (thizaole-O-*C*-N), 155.74 (N-*CH*-S), 155.53 (Val-NH-*C*(O)-O), 152.07 (Val-NH-C(O)-O -CH_2_-*C*), 150.58 (**C**-N-Et_2_), 143.21 (S-C-*CH*-N), 139.640 (i*-Ph*-CH_2_-CH-NH-thiazol), 138.86 (i-*Ph*-CH_2_-CH-NH-Val), 134.29 (S-*C*-CH-N), 129.19 and 129.52 (2 × o-*Ph*), 128.18 and 128.03 (2 × m-*Ph*), 126.00 and 125.94 (2 × p-*Ph*), 125.50 (C-*CH*-CH-C-N-Et_2_), 108.91 (C-CH-*CH*-C-N-Et_2_), 105.43 (**C**-CH-CH-C-N-Et_2_), 104.63 (O-C(O)-*CH*-C), 97.05 (C-*CH*-C-N-Et_2_), 69.15 (HO-*CH*), 61.16 (O-*CH*_*2*_-coumarin), 60.57 (NH-C(O)-*CH*(iPr)-NH), 57.37 (C(O)O-*CH*_*2*_-thiazole), 55.73 (NH-*CH***-**CH-OH), 47.30 (OH-CH-CH_2_-*CH*-NH), 44.20 (*CH*_*2*_-CH_3_), 39.53 (Val-NH-CH-*CH*_*2*_-Ph), 38.29 (OH-CH-*CH*_*2*_-CH-NH), 37.34 (*CH*_*2*_-CH-NH-C(O)o-thiazole), 30.55 (*CH*(CH_3_)_2_), 19.43 and 18.33 (2 × *CH*_*3*_), 12.50 (CH_2_-*CH*_*3*_). HRMS (m/z; ESI+): calculated for C_43_H_51_O_8_N_5_S [MNa]^+^ 820.33506; found 820.33470.

### Purification of HIV PR

The recombinant PR was overexpressed in *Escherichia coli* BL21(DE3) RIL (Novagen). Protein expression and isolation of inclusion bodies were carried out as previously described^34^. Inclusion bodies were solubilized in 67% (v/v) acetic acid and refolded by dilution into a 25-fold excess of water, followed by overnight dialysis at 4 °C against water and then against 50 mM MES (pH 5.8), 10% (v/v) glycerol, 1 mM EDTA and 0.05% (v/v) 2-mercaptoethanol. The PR was purified by cation exchange chromatography using MonoSFPLC (Pharmacia). The enzyme was stored at 70 °C until further use^34^.

### Inhibition of HIV-1 PR

*K*_i_ values were determined by spectrophotometric assay using purified recombinant HIV-1 PR and the chromogenic substrate KARVNleNphEaNle-NH_2_. Data were analyzed using the Morrison equation^35^.

### Crystallization experiments

The HIV PR-compound **1** complex was prepared by mixing the enzyme with an equimolar amount of **1** dissolved in DMSO. The protein was pre-concentrated to 4 mg ml^−1^ by ultrafiltration using Microcon-10 filters (Millipore, Billerica, MA, USA). The complex was then centrifuged for 25 min at 16,000 *g* to reduce the number of crystallization nuclei. Crystals were then grown by the hanging drop vapour diffusion technique at 19 °C. The crystallization drops contained 2 μl protein-inhibitor complex and 1 μl reservoir solution (0.2 M lithium sulfate, 0.1 M phosphate/citrate pH 4.2 and 20% (w/v) PEG 1000; JSCG+ condition 6). The HIV PR-compound **2** complex was prepared by incubation HIV PR with fourfold molar excess of **2** for 30 min and it was then concentrated to a protein concentration of 4 mg ml^−1^ by the above described procedure. Crystals were grown as described above. The reservoir solution was 0.2 M magnesium chloride, 0.1 M Tris pH 8.5 and 20% (w/v) PEG 8000. For diffraction measurements, crystals were soaked in reservoir solution supplemented with 25% (v/v) glycerol and cooled in liquid nitrogen. The diffraction data collection and structure refinement are described in Supplementary Information in chapter 3.3.

### Photolysis of the inhibitor

Irradiation was performed in two distinct set ups.

*Cuvette set up*. A 1 ml reaction mixture (8 nM purified recombinant HIV-1 PR in cleavage buffer—100 mM sodium acetate, 0.3 M NaCl, 4 mM EDTA, pH 4.7, various concentrations of inhibitor) at 4 °C was irradiated with two defocused 405 nm lasers (130 and 170 mW) in a quartz cuvette for various periods of time. The cuvette was then equlibrated to 37 °C, and the enzymatic reaction was started by adding 4 μl of 3.8 μM chromogenic substrate (KARVNleNphEaNle-NH_2_)^27^. The enzyme activity (and thus the efficacy of photodegradation) was followed by the decrease in absorbance at 305 nm.

*Capillary set up*. Two 405 nm focusable lasers (130 and 170 mW, checked for both intensity and wavelength before use) were used for irradiation in a capillary set up. The solution (either purified HIV-1 PR or a suspension of immature virions) was linearly pumped through a 250 μm glass capillary (Hirschmann ring caps) at which both laser beams were focused (for an illustrative photo, see Supplementary Fig. 4). To 47.5 μl of cleavage buffer (100 mM sodium acetate, 0.3 M NaCl, 4 mM EDTA, pH 4.7) on ice, 2 μl of 4 μM HIV-1 PR and 0.5 μl of 200 μM compound **1** were added. The solution was irradiated at different flow rates, diluted in a 1 ml cuvette with 950 μl of cleavage buffer (100 mM sodium acetate, 0.3 M NaCl, 4 mM EDTA, pH 4.7, 37 °C) and the enzymatic reaction was started by adding 4 μl of 3.8 μM chromogenic substrate.

### Analysis of photodegradation products

The photodegradation of the inhibitor was analyzed with an analytical Jasco PU-1580 HPLC (flow rate 1 ml min^−1^, invariable gradient 2–100% ACN in 30 min, Watrex C18 Analytical Column, 5 μm, 250 × 5 mm), and the retention times were compared with those of synthetic standards (the degradation product was also an intermediate during synthesis of compound **1**).

### Plasmids and cell cultures

Proviral plasmid pNL4-3 (obtained through the NIH AIDS Reagent Program from Dr Malcolm Martin) has been described before^36^. Plasmid pCHIV, which encodes all HIV-1 NL4-3 proteins except Nef, but lacks both long terminal repeat regions required for infectivity, has also been described^37^. HEK293T cells were kept in high-glucose Dulbecco’s modified Eagle’s medium (DMEM, Life Technologies) supplemented with penicillin/streptomycin and 10% foetal calf serum at 37 °C, 5% CO_2_. For the analysis of inhibitor activity on viral particle processing, HEK293T cells were transfected with pNL4-3 using calcium phosphate, and inhibitor was added at the indicated concentrations. At 48 h post transfection, supernatants were harvested, cleared by filtration through a 0.45-μm filter and concentrated by ultracentrifugation through a 20% (w/w) sucrose cushion.

### Photoactivation of HIV-1 PR *in situ*

HEK293T cells were seeded in six-well plates in high-glucose, phenol red-free DMEM supplemented with penicillin/streptomycin and 10% foetal calf serum. On the following day, cells were transfected with plasmid pCHIV^37^ using polyethyleneimine according to standard procedures. A final concentration of 2 μM compound **1** or DMSO (vehicle) was added to the tissue culture medium at the time of transfection. At 44 h post transfection, tissue culture supernatants were harvested, adjusted to pH 6.0 using PR buffer (50 mM MES, pH 6.0, 150 mM NaCl, 2 mM DTT, 1 mM EDTA). Alternatively, supernatants were harvested, filtered, inhibitor was removed by two successive ultracentrifugation steps through a 20% sucrose cushion and the particle pellet was resuspended in PR buffer. In both cases, samples were then split into two aliquots, one of which was subjected to ultraviolet irradiation using the capillary set up described above, whereas the control aliquot was pumped through the capillary set up without ultraviolet irradiation. Subsequently, samples were incubated at 37 °C, and 20 μl aliquots were taken before incubation (*t*=0) and at *t*=15, 30, 60, 90 and 360 min. At these time points, the processing reaction was stopped by addition of SDS sample buffer and heat treatment (5 min, 90 °C).

### Analysis of HIV polyprotein processing by immunoblot

Samples were separated by SDS–PAGE (17.5%; acrylamide:bisacrylamide 200:1), and proteins were transferred to a nitrocellulose membrane by semi-dry blotting. HIV-1 Gag-derived proteins were detected using rabbit polyclonal antiserum raised against HIV-1 CA, followed by fluorescently labelled goat-anti rabbit secondary antibody (LiCor). Quantification of anti-CA reactive bands was performed using an infrared imaging system (LiCor Odyssey) and Image Studio Lite software. Data were analyzed with GraphPad prism.

## Author contributions

J.S. designed the compounds; J.S. with P.M. and P.C. synthesized the compounds; J.S. evaluated the compounds in vitro; J.W., M.P., M.A. and B.M. analyzed the antiviral activity and inhibition of polyprotein processing; J.S. and P.Š. designed the irradiation apparatus; J.S., P.P. and P.Ř. crystallized the PR complexes and solved the structures; M.A., J.S., B.M. and H.-G.K. analyzed the polyprotein processing by photoactivation; J.K. conceived and lead the project; J.S., J.K., H.-G.K. and B.M. analyzed the data, J.S., H.-G.K., P.Ř., B.M. and J.K. wrote the manuscript.

## Additional information

**How to cite this article:** Schimer, J. *et al*. Triggering HIV polyprotein processing by light using rapid photodegradation of a tight-binding protease inhibitor. *Nat. Commun.* 6:6461 doi: 10.1038/ncomms7461 (2015).

**Accession codes**: Atomic coordinates and experimental structure factors have been deposited in the Protein Data Bank under codes 4U7Q and 4U7V for complexes with compounds **1** and **2**, respectively

## Supplementary Material

Supplementary Information Supplementary Figures 1-5, Supplementary Table 1, Supplementary Methods and Supplementary References
