# Supplementary material for: Triggering HIV polyprotein processing by light using rapid photodegradation of a tight-binding protease inhibitor
Source: Nat Commun. 2015 Mar 9;6:6461. doi: 10.1038/ncomms7461 (PMC4366505; doi:10.1038/ncomms7461)
Supplement: Supplementary Information — Supplementary Figures 1-5, Supplementary Table 1, Supplementary Methods and Supplementary References [file ncomms7461-s1.pdf]

1. Supplementary figures

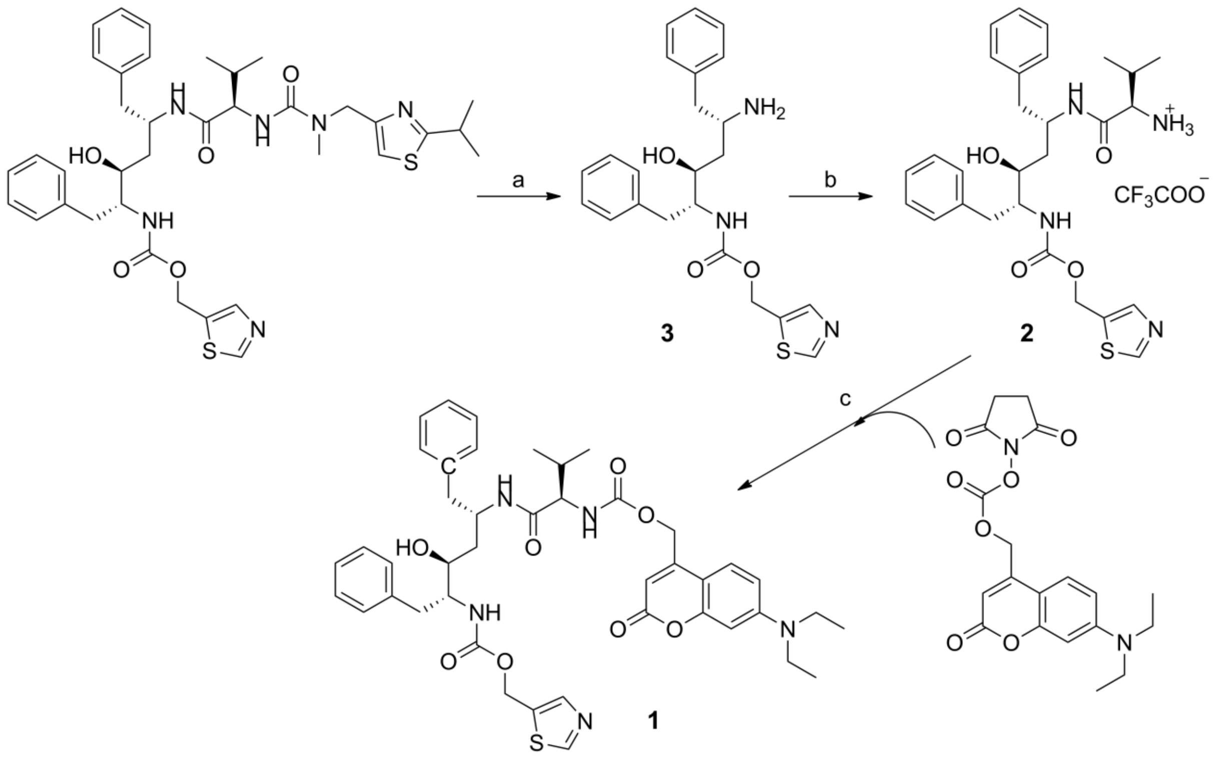

a) Dioxan/HCl, 20h 65°C; b) 1) Boc-Val-OH, TBTU, DIEA, DMF 2)TFA; c) DIEA, DMF

Supplementary Figure 1. Synthesis of 1

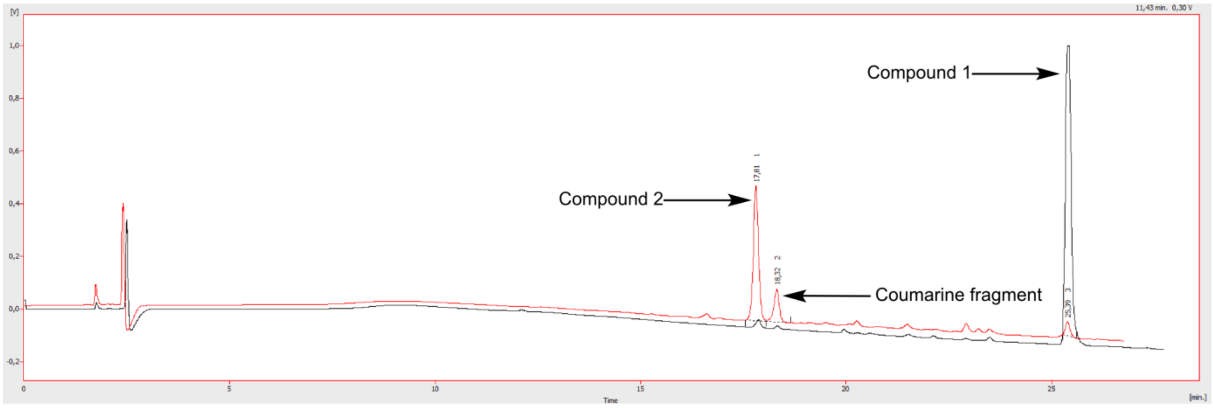

Supplementary figure 2: **HPLC determination of 1 photodegradation:** Degradation of **1** determined by analytical HPLC using Watrex C18 Analytical Column, 5 µm, 250 x 5 mm and 2-100 % acetonitrile gradient in 30 min. The black chromatograph refers to a non-irradiated sample, the red one to

irradiated sample. The retention time of all fragments was checked with the retention times of purified standards.

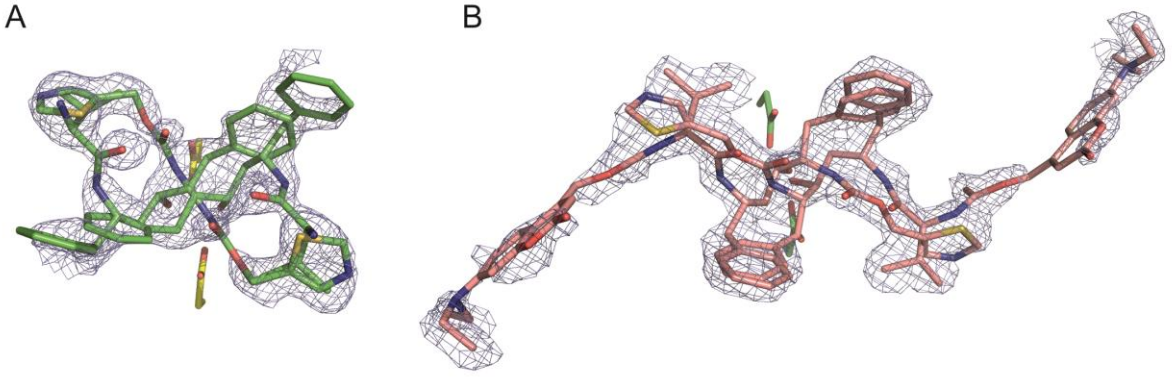

Supplementary figure 3 Active center with inhibitor: Details of compound **1**(**A**) and **2** (**B**) bound to HIV PR. The 2*Fo-Fc* electron density maps contoured at 1.0 σ. Two alternative inhibitor positions related by 180º rotation with 50% relative occupancy are shown in sticks. Catalytic aspartates are also shown as sticks.

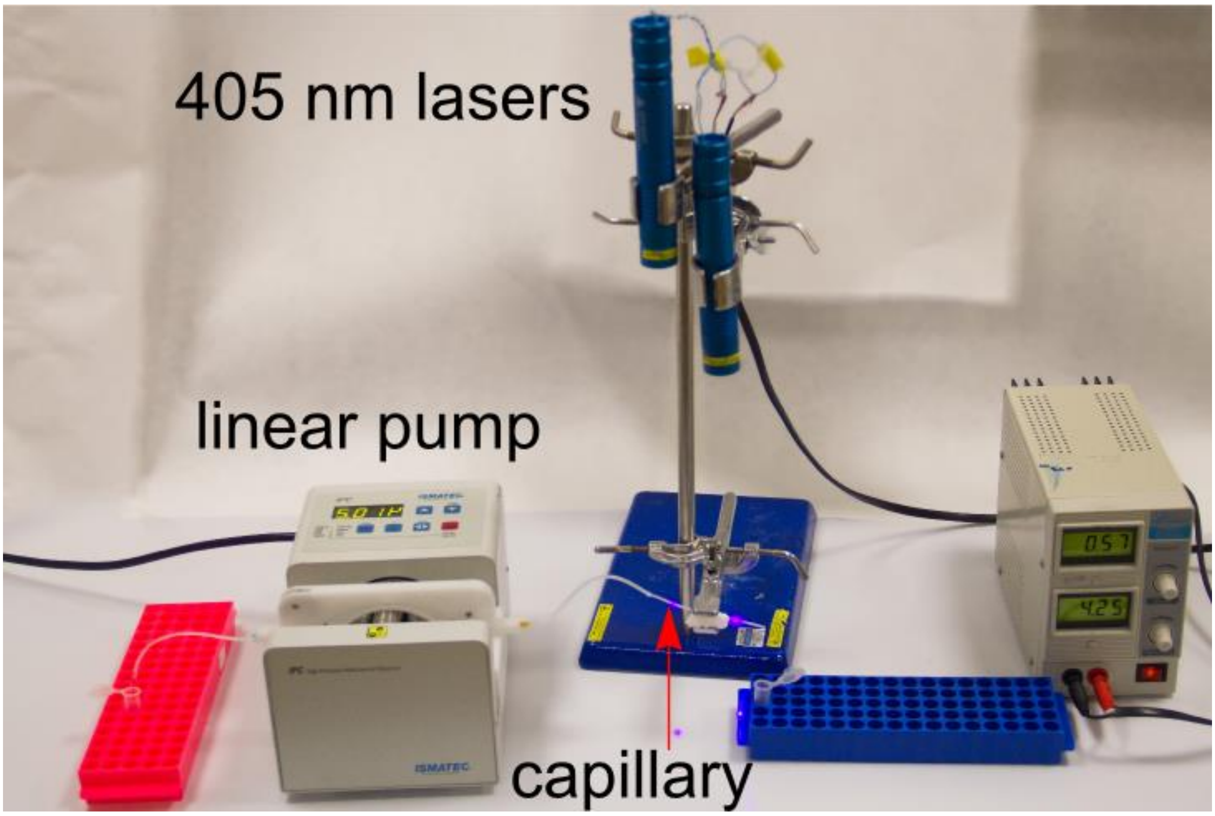

Supplementary figure 4. **Capillary setup** A capillary setup used for degradation of photolabile inhibitors. This setup proved to be far superior to other setups tested.

A

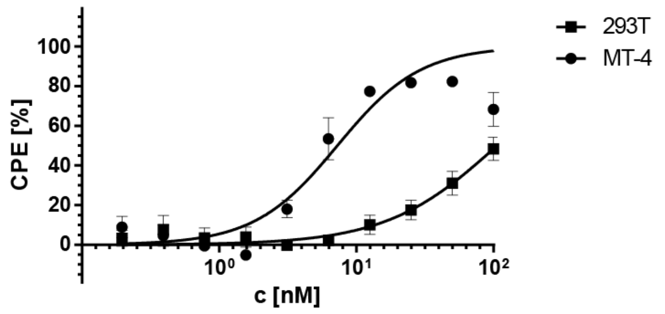

B

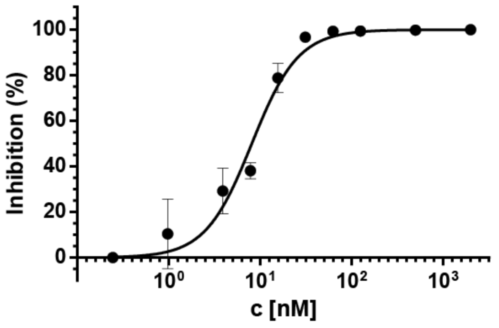

Supplementary figure 5 Cytotoxicity of 1.(**A**) Cytotoxicity of **1** in HEK293T and MT-4 cells (CC_{50}>50 µM and 7.3 µM, respectively), expressed as cytopathic effect (CPE); (**B**) inhibition of HIV-1 by **1** in MT-4 cells (EC_{50} =8.1 nM).

2. Supplementary Tables

Supplementary Table 1 Crystalographic data: Data collection and model refinement statistics for structural analysis of HIV PR complexes with **1** and **2**

**Data collection statistics**		
Compound	**1**	**2**
PDB code	4U7Q	4U7V
Space group	$P6_1$	$P6_1$
Cell parameters (Å; °)	a = b = 62.65, c = 80.12; α= β = 90, γ= 120	a = b =62.6, c = 82.06; α= β = 90, γ= 120
Number of molecules in AU	2	2
Wavelength (Å)	0.9184	0.9184
Resolution (Å)	44.92-1.61 (1.71-1.61)	-1.38 (1.46-1.38)

Number of unique reflections	23,062 (3,707)	35441 (4,447)
Multiplicity	5.1 (5.0)	4.7 (4.3)
Completeness (%)	99.7 (99.8)	95.8 (94.1)
R_{meas}[a]	4.5 (71.1)	4.9 (75.7)
Average $I/\sigma(I)$	20.8 (2.57)	17.8 (2.15)
Wilson B (Å^2)[b]	31.48	23.66
**Refinement statistics**		
Resolution range (Å)	10.0-1.70	32.72 -1.38
No. of reflections in working set	18,654 (1,253)	33,658 (1,711)
No. of reflections in test set	978 (61)	1,772 (90)
R value (%)[c]	27.5 (34.6)	20.8 (27.9)
R_{free} value (%)[d]	32.6 (35.4)	24.7 (31.3)
RMSD bond length (Å)	0.016	0.027
RMSD angle (°)	2.191	2.518
Number of atoms in AU	1,701	1,830
Number of protein atoms in AU	1,494	1,562
Number of water molecules in AU	93	192
Mean B value (Å^2)	34.9	22.2
Ramachandran plot statistics[e]		
Residues in favored regions (%)	95.5	97.2
Residues in allowed regions (%)	4.0	2.8

The numbers in parentheses refer to the highest-resolution shell.

[a] $R_{meas} = \Sigma_{hkl}\{N(hkl)/[N(hkl) -1]\}^{1/2} \Sigma_i |I_i(hkl)-\langle I(hkl)\rangle| / \Sigma_{hkl} \Sigma_i I_i(hkl)$ where $\langle I(hkl)\rangle$ is the mean of the N(*hkl*) individual measurements $I_i(hkl)$ of the intensity of reflection *hlk* (Diederichs & Karplus, 1997).

[b] Wilson B calculatedby Sfcheck program from CCP4 suite.[5]

[c] R-value = $||F_o| - |F_c|| / |F_o|$, where F_o and F_c are the observed and calculated structure factors, respectively.

[d] R_{free} is equivalent to R value but is calculated for 5% of the reflections chosen at random and omitted from the refinement process.[11]

[e]as determined by MolProbity.[9]

3. Supplementary Methods

3.1 Chemical synthesis

All chemicals were purchased from Sigma-Aldrich, unless stated otherwise. All inhibitors tested in the biological essays were purified using preparative scale HPLC Waters Delta 600 (flow rate 7 ml/s, gradient shown for each compound - including R_t) with column Waters SunFire C18 OBD Prep Column, 5 µm, 19 x 150 mm. The purity of compounds was tested on analytical Jasco PU-1580 HPLC (flow rate 1 ml/s, invariable gradient 2-100 % ACN in 30 minutes, R_t shown for each compound) with column Watrex C18 Analytical Column, 5 µm, 250 x 5 mm. The final inhibitors were all at least of 99 % purity. Structure was further confirmed by HRMS at LTQ Orbitrap XL (Thermo Fisher Scientific) and by NMR (Bruker Avance I™ 500 M equipped with Cryoprobe or Bruker Avance I™ 400 M). All peaks in NMR spectra for all compounds were assigned using standard 2D NMR techniques (COSY, HMBC, HSQC).

Isolation of ritonavir (RTV) from commercially available capsules: RTV is suspended in capsules in an oily mixture of rather non–polar compounds. 50 tablets (100 mg RTV each) were cut open and the oily substance was squeezed out into a bottom round shaped 2 l flask. 200 ml of hexane was added along with 500 ml of diethylether. The resulting suspension was triturated and sonicated for 3 hours until all oil turned into a white precipitate. This precipitate was filtred and again triturated/sonicated in pure diethylether, after which the pure RTV was filtered. 3.6 g of RTV was obtained (yield 72 %). The purity of RTV was determined by HPLC and was well above 99 %.

Partial hydrolysis of ritonavir (RTV), thiazol-5-ylmethyl ((*2R,3S,5S*)-5-amino-3-hydroxy-1,6-diphenylhexan-2-yl)carbamate, compound **3**: 1.00 g of RTV was dissolved in 50 ml of dioxane in a bottom round flask. 50 ml of concentrated hydrochloric acid was added and the resulting mixture was stirred at 65 °C for 20 hours (note that different temperature and/or time lead to different cleavage products). After 20 hours the mixture was let to cool down to RT. The mixture was neutralized by addition of K_2CO_3 until the resulting mixture showed basic pH. The solvents were concentrated using rotary evaporater to roughly 50 ml and diluted by 150 ml of water and washed 3 times by 100 ml of EtOAc. The water phase was discarded and organic phase was dried and evaporated. 885 mg of crude mixture was obtained and was used in the next reaction without further purification (purity roughly 80 % - HPLC determination). For spectral determination, 50 mg were purified using preparative HPLC (gradient: 20 %-50 % ACN in 40 minutes. R_t 15 minutes); Analytical HPLC R_t = 17.3 min. HRMS (ESI+): calculated for $C_{23}H_{28}O_3N_3S$ $[M]^+$ 426.18459. Found 426.18454. ^{1}H NMR (500 M, DMSO-*d6*): δ 9.06 (d, ^4J=0.8, 1H, N-*CH*-S), 7.84 (q, ^4J=0.8, 1H, S-C-*CH*-N), 7.81 (bs, 3H, NH_3^+), 7.32-7.15 (m, 10H, *Ph*-), 7.20 (bs, 1H, *NH*), 5.50 (bs, 1H, *OH*), 5.15 (dd, J_{gem}=13.2 , ^4J=0.8 , 1H, O-CH_2), 5.11 (dd, J_{gem}=13.2 , ^4J=0.8 , 1H, COO-CH_2), 3.69 (m, 1H, HO-*CH*), 3.67 (m, 1H, HO-CH-*CH*-NH), 3.50 (bm, 1H, NH_3^+-*CH*), 2.87 (dd, J_{gem}=14.0 , *J*=6.4 , 1H, NH_3^+-CH-CH_2-Ph), 2.80 (dd, J_{gem}=14.0 , *J*=7.3 , 1H, NH_3^+-CH-CH_2-Ph), 2.79 (dd, J_{gem}=13.7 , *J*=3.7 , 1H, NH-CH-CH_2-Ph), 2.79 (dd, J_{gem}=13.7 , *J*=10.5 , 1H, NH-CH-CH_2-Ph), 1.58 (bs, 2H, OH-CH-CH_2-CH). ^{13}C NMR (125.7 M, DMSO-*d6*): δ 155.77 (N-*CH*-S), 155.39 (O-*C*-N), 143.23 (S-C-*CH*-N), 139.52 (*Ph*), 136.37 (*Ph*), 134.14 (S-*C*-CH-N), 129.61 (*Ph*), 129.18 (*Ph*), 128.81 (*Ph*), 128.23 (*Ph*), 127.07 (*Ph*), 126.12 (*Ph*), 69.81 (HO-*CH*), 57.49 (COO-CH_2), 56.94 (HO-CH-*CH*-NH), 50.87 (NH_3^+-*CH*), 38.71 (NH_3^+-CH-CH_2-Ph), 35.69 (NH-CH-CH_2-Ph), 34.66 (CH-CH_2-CH).

Thiazol-5-ylmethyl ((*2R,3S,5S*)-5-((*R*)-2-amino-3-methylbutanamido)-3-hydroxy-1,6-diphenylhexan-2-yl)carbamate, Compound **2**: 526 mg of TBTU (1.64 mmol, 1.0 eq) was added to 356 mg BOC-Val-OH (1.64 mmol, 1.0 eq) dissolved in 1.5 ml of DMF along with 690 µl of DIEA (3.94 mmol, 2.4 eq). The crude hydrolysate of RTV (700 mg, 1.64 mmol, 1 eq), dissolved in 1 ml of DMF, was added after 5 minutes of stirring in one portion. The reaction was left overnight and the DMF was rotary evaporated. The reaction mixture was dissolved in 50 ml of EtOAc and washed two times by saturated $NaHCO_3$, two times by 10% $KHSO_4$ and once with brine. The organic mixture was dried, evaporated and the product was purified using Flash chromatography (TLC analysis: EtOAc, R_f= 0.65). Product was further dissolved in 5 ml of hot EtOAc and 5 ml of diethyl ether were added. The resulting gel was filtrated and dried to give very pure (>99 %, HPLC determination) 250 mg of product (yield 25 %). Analytical HPLC R_t = 17.4 min. HRMS (ESI+): calculated for $C_{28}H_{37}O_4N_4S$ $[M]^+$ 525.25300. Found 525.25292.^{1}H NMR (500 M, DMSO-*d6*): δ 9.06 (d, ^4J=0.8 , 1H, N-*CH*-S), 8.24 (d, *J*=8.2 , 1H, -*NH*-CO), 8.00 (bd, *J*=5.2 , 3H, -NH_3^+), 7.85 (q, ^4J=0.8 , 1H, S-C-*CH*-N), 7.28-7.13 (m, 10H, *Ph*-), 6.94 (d, *J*=9.4 , 1H, *NH*-CO-O), 5.12 (d, ^4J=0.8 , 2H, O-CH_2), 4.16 (m, 1H, *CH*-NH-CO), 3.78 (m, 1H, *CH*-NH_3^+, partial overlap with water residual peak), 3.58 (td, *J*=6.8 , *J*=2.0 , 1H, *CH*-OH), 3.48 (m, 1H, Ph-CH2-*CH*-NH),

2.72-2.67 (m, 4H, 2xCH-*CH₂*-Ph), 2.00 (m, 1H, *CH*-$(CH_3)_2$), 1.50 (m, 1H, OH-CH-*CH₂*), 1.43 (m, 1H, OH-CH-*CH₂*), 0.89 (d, *J*=6.8 , 3H, -*CH₃*), 0.84 (d, *J*=6.8 , 3H, -*CH₃*). ^{13}C NMR (125.7 M, DMSO-*d6*): δ 167.33 (*CO Val*), 158.33(q, $J_{C,F}$=34.4, CF_3*COO*-), 155.79 (O-C-N), 155.71 (N-*CH*-S), 143.23 (S-C-*CH*-N), 139.50 (*Ph*), 138.55 (*Ph*), 134.23 (S-*C*-CH-N), 129.56 (*Ph*), 129.17 (*Ph*), 128.30 (*Ph*), 128.25 (*Ph*), 126.26 (*Ph*), 126.09 (*Ph*), 116.44 (q $J_{C,F}$= 294.8, CF_3-COO^-) 68.90 (HO-*CH*), 57.56 (CO-*CH*-NH_3), 57.44 (COO-*CH₂*), 55.74 (HO-CH-*CH*-NH), 47.98 (CONH-*CH*), 39.75 (NH-CH-*CH₂*-Ph), 37.77 (-CH_2-*CH*-CH-), 37.33 (Ph-*CH₂*-CH-NH), 30.04 (*CH*$(CH_3)_2$), 17.26 and 18.69 (2x*CH₃*).

(7-(diethylamino)-2-oxo-2H-chromen-4-yl)methyl (2,5-dioxopyrrolidin-1-yl) carbonate: 210 mg of 7-(diethylamino)-4-(hydroxymethyl)-2H-chromen-2-one (0.85 mmol, 1.0 eq) was dissolved in 4 ml of DCM along with 237 µl of DIEA (1.36 mmol, 1.6 eq). DSC (326 mg, 1.27 mmol, 1.5 eq) was added in one portion and the reaction was left stirring overnight. The volatiles were then evaporated and the crude mixture was purified by column chromatography on silica (He:EtOAc: 1:1; R_f=0.2). Note: the product has almost same R_f as the reactant, but gives slightly different colour when treated with phosphomolybdic solution. The product is not very stable and is hydrolyzed even in freezer when any moisture is present over longer period of time (e.g. 2 months). 120 mg (yield=37 %) of yellow oil (turns crystalline when the last traces of solvents were removed) was obtained. Purity 90 % (contaminated by coumarine reactant). HRMS (ESI+): calculated for $C_{19}H_{20}O_7N_2$ $[MH]^+$ 389.13433. Found 389.13424. ^1H NMR (500 M, DMSO-d6) δ 7.46 (d, *J* = 9.1, 1H), 6.73 (dd, *J* = 9.1, 2.5 , 1H), 6.57 (d, *J* = 2.5 , 1H), 6.01 (s, 1H), 5.63 (s, 2H), 3.44 (d, *J* = 7.0 , 4H), 2.83 (s, 4H), 1.12 (t, *J* = 5.7 , 6H). ^{13}C NMR (126 M, DMSO-d6) δ 170.02, 160.58, 157.08, 156.11, 151.23, 150.86, 148.49, 125.83, 109.07, 105.98, 105.04, 97.10, 67.88, 59.23, 44.22, 25.59, 12.50.

3.2 Photodegradation of 1 determined by HPLC:

10 µM solution of **1** in distilled water was irradiated by lasers at 15 µl/min flow rate and the products were then extracted by ethyl acetate (EtOAc). The EtOAc was then evaporated, the residual film was dissolved in 20 µl of acetonitrile and the sample composition was determined using analytical HPLC (Figure 2).

3.3 Crystallographic analysis

Diffraction data were collected at 100 K at beamlines BL14.2 of BESSY, Berlin, Germany[2] at wavelength of 0.9184 Å. Diffraction data were integrated and reduced using MOSFLM[3] and scaled using SCALA[4] from the CCP4 suite.[5] Crystals exhibited $P6_12$ symmetry and contained one HIV PR dimer in the asymmetric unit. Crystal parameters and data collection statistics are given in Table 1.

The structure was determined by molecular replacement using the program Molrep[6] with the structure of HIV PR the template [PDB code 1U8G]. Initial model refinement was carried out using the program REFMAC 5.2[7] from the CCP4 package,[5] interspersed with manual adjustments using Coot.[8]

The quality of the final models was validated with Molprobity.[9] Refinement statistics are given in Table S1. All figures showing structural representations were prepared with the program PyMOL.[10] Atomic coordinates and experimental structure factors have been deposited in the Protein Data Bank under codes 4U7Q and 4U7V for complexes with **1** and **2**, respectively.

3.4 Inhibition potency and cytotoxicity of 1

Cytotoxicity was determined by incubating two-fold serial dilutions of **1** in triplicate in a 96-well plate with 30,000 HEK293T cells/well seeded day ago in Dulbecco's modified Eagle's medium with L-glutamine supplemented with 10% fetal calf serum (FCS, PAA), 100 U of penicillin/mL, 100 µg of streptomycin/mL (Sigma-Aldrich) and with 30,000 MT-4 cells (obtained through the NIH AIDS Reagent Program, Division of AIDS, NIAID, NIH from Dr. Douglas Richman) seeded in RPMI 1640 medium with 2 mM L-glutamine (PAA) supplemented with 10% FCS, 10 mM HEPES (Sigma-Aldrich), 100 U of penicillin/ml, and 100 µg of streptomycin/mL. After 72 h of incubation at 37 °C, 5 % CO_2, tetrazolium salt XTT (Sigma-Aldrich) was added to the wells for 4 h and formation of colored formazan solution was quantified using Victor X3 plate reader (Perkin Elmer). The percentage of cytopathic effect (CPE) was plotted against the $\log_{10}$ **1** concentrations and the 50 % cytotoxic concentrations (CC_{50}) were calculated using nonlinear regression analysis with GraphPad Prism ver. 6 (GraphPad Software). The anti-HIV-1 activity of **1** was determined by infecting MT-4 cells with HIV-$1_{NL4\text{-}3hRluc}$ (HIV expressing human *Renilla* luciferase[12]) at multiplicity of infection 0.001 IU/ml for 1 h at 37 °C, 5 % CO_2 and plating 30,000 infected cells/well in a 96-well plate with pre-plated two-fold serial dilutions of **1**. After 72 h incubation at 37 °C, 5 % CO_2 HIV-1 activity was quantified by measuring *Renilla* luciferase luminescence using the *Renilla* Luciferase Assay System (Promega) in Victor X3 plate reader. Percentage of inhibition of luminescence activity was plotted against the $\log_{10}$ **1** concentrations and the 50 % effective concentration (EC_{50}) was calculated using nonlinear regression analysis with GraphPad Prism software.

4. Supplementary References

1. Copeland, R.A. *Enzymes: A Practical Introduction to Structure, Mechanism, and Data Analysis*, (Wiley-VCH, New York, 2000).
2. Mueller, U. et al. Facilities for macromolecular crystallography at the Helmholtz-Zentrum Berlin. *J Synchrotron Radiat* **19**, 442-449 (2012).
3. Leslie, A.G. Integration of macromolecular diffraction data. *Acta Crystallogr D Biol Crystallogr* **55**, 1696-1702 (1999).
4. Evans, P. Scaling and assessment of data quality. *Acta Crystallogr D Biol Crystallogr* **62**, 72-82 (2006).
5. CCP4. The CCP4 suite: programs for protein crystallography. *Acta Crystallogr D Biol Crystallogr* **50**, 760-763 (1994).
6. Vagin, A. & Teplyakov, A. An approach to multi-copy search in molecular replacement. *Acta Crystallogr D Biol Crystallogr* **56**, 1622-1624 (2000).
7. Murshudov, G.N. et al. REFMAC5 for the refinement of macromolecular crystal structures. *Acta Crystallographica Section D-Biological Crystallography* **67**, 355-367 (2011).
8. Emsley, P. & Cowtan, K. Coot: model-building tools for molecular graphics. *Acta Crystallogr D Biol Crystallogr* **60**, 2126-2132 (2004).
9. Lovell, S.C. et al. Structure validation by Calpha geometry: phi,psi and Cbeta deviation. *Proteins* **50**, 437-450 (2003).
10. DeLano, W.L. The PyMOL Molecular Graphics System. DeLano Scientific LLC, San Carlos, CA, USA. http://www.pymol.org). (2002).

11. Brunger, A.T. Free R-Value - a Novel Statistical Quantity for Assessing the Accuracy of Crystal-Structures. *Nature* **355**, 472-475 (1992).
12. Weber, J. et al. Novel method for simultaneous quantification of phenotypic resistance to maturation, protease, reverse transcriptase, and integrase HIV inhibitors based on 3'Gag(p2/p7/p1/p6)/PR/RT/INT-recombinant viruses: a useful tool in the multitarget era of antiretroviral therapy. *Antimicrob Agents Chemother* **55**, 3729-3742 (2011).
